# Walking Speed of Children and Adolescents With Cerebral Palsy: Laboratory Versus Daily Life

**DOI:** 10.3389/fbioe.2020.00812

**Published:** 2020-07-14

**Authors:** Lena Carcreff, Corinna N. Gerber, Anisoara Paraschiv-Ionescu, Geraldo De Coulon, Kamiar Aminian, Christopher J. Newman, Stéphane Armand

**Affiliations:** ^1^Laboratory of Kinesiology Willy Taillard, Geneva University Hospitals, University of Geneva, Geneva, Switzerland; ^2^Pediatric Neurology and Neurorehabilitation Unit, Department of Pediatrics, Lausanne University Hospital, Lausanne, Switzerland; ^3^Laboratory of Movement Analysis and Measurement, Ecole Polytechnique Fédérale de Lausanne, Lausanne, Switzerland; ^4^Pediatric Orthopedics, Geneva University Hospitals, Geneva, Switzerland

**Keywords:** cerebral palsy, typical development, capacity, performance, inertial sensors, walking speed

## Abstract

The purpose of this pilot study was to compare walking speed, an important component of gait, in the laboratory and daily life, in young individuals with cerebral palsy (CP) and with typical development (TD), and to quantify to what extent gait observed in clinical settings compares to gait in real life. Fifteen children, adolescents and young adults with CP (6 GMFCS I, 2 GMFCS II, and 7 GMFCS III) and 14 with TD were included. They wore 4 synchronized inertial sensors on their shanks and thighs while walking at their spontaneous self-selected speed in the laboratory, and then during 2 week-days and 1 weekend day in their daily environment. Walking speed was computed from shank angular velocity signals using a validated algorithm. The median of the speed distributions in the laboratory and daily life were compared at the group and individual levels using Wilcoxon tests and Spearman’s correlation coefficients. The corresponding percentile of daily life speed equivalent to the speed in the laboratory was computed and observed at the group level. Daily-life walking speed was significantly lower compared to the laboratory for the CP group (0.91 [0.58–1.23] m/s vs 1.07 [0.73–1.28] m/s, *p* = 0.015), but not for TD (1.29 [1.24–1.40] m/s vs 1.29 [1.20–1.40] m/s, *p* = 0.715). Median speeds correlated highly in CP (*p* < 0.001, rho = 0.89), but not in TD. In children with CP, 60% of the daily life walking activity was at a slower speed than in-laboratory (corresponding percentile = 60). On the contrary, almost 60% of the daily life activity of TD was at a faster speed than in-laboratory (corresponding percentile = 42.5). Nevertheless, highly heterogeneous behaviors were observed within both populations and within subgroups of GMFCS level. At the group level, children with CP tend to under-perform during natural walking as compared to walking in a clinical environment. The heterogeneous behaviors at the individual level indicate that real-life gait performance cannot be directly inferred from in-laboratory capacity. This emphasizes the importance of completing clinical gait analysis with data from daily life, to better understand the overall function of children with CP.

## Introduction

The World Health Organization (WHO) has emphasized the need to consider both capacity, defined as what a person can do in a standardized environment, and performance, defined as what a person does in his/her habitual environment, to describe a person’s activity. These descriptors account for the role of impairments in body functions as well as the impact of the environment and other personal factors on activity and participation (World Health Organization, 2002). Since the usual environment includes the overall societal context, performance considers external and personal factors, unlike capacity, which focuses on functional abilities. Walking capacity and performance can thus be interpreted as walking in clinical (laboratory) and daily life settings, respectively, (Bjornson, 2019).

In children with cerebral palsy (CP), gait capacity assessments are a mainstay of clinical evaluation (Gerber et al., 2019). These children, who present lifelong motor disabilities (O’Shea, 2008), are regularly assessed in clinical settings through diverse functional tests, such as the Gross Motor Function Measure (GMFM) (Alotaibi et al., 2014) and the 6-Min Walk Test (Enright, 2003), or through an exhaustive assessment of gait deviations using 3D clinical gait analysis (Armand et al., 2016; Carcreff et al., 2016). This data on gait capacity is used to identify, quantify and understand the motor disorders, and serves as a support for the management of gait deviations (Armand et al., 2016). However, walking under the supervision of the clinician in a laboratory may not always be representative of usual walking (Gosselin et al., 2018). The patient’s capacity is usually overestimated as he/she shows the best of him/herself to please the care providers (Toosizadeh et al., 2015), a phenomenon often referred to as the ‘Hawthorne effect’ (Berthelot et al., 2011). Additional information about the patient’s unsupervised walking (i.e., daily life-based gait performance), as a complement to laboratory-based assessments, could improve the understanding of the patient’s overall gait difficulties, enhancing clinical care (Young et al., 1996; Bjornson et al., 2013; Warmerdam et al., 2019). It is meaningful for treatment objectives to determine whether the effect of a treatment, observed in clinical settings by improvements in capacity, generalizes into the patient’s daily life by improving performance. Recently Halma et al. (2020) have demonstrated that improvements in motor capacity are largely not accompanied by changes in motor performance in ambulatory children with CP within the context of intensive therapy, and have emphasized the need to include interventions specifically aimed at improving motor performance into treatment programs. In addition, by providing a high amount of quantitative data about the patient’s habitual gait and confronting it with clinic-based observations it could help to determine the level of confidence with which clinicians might predict gait performance based on laboratory-based assessments. The other way around, patients might improve their daily functioning by simply adapting the environment (World Health Organization, 2002), in absence of any change in capacity. Thus, if proven to be efficient (i.e., using objective performance measurement tools), clinicians might focus on adapting the environment and working on personal factors (e.g., with self-efficacy training) especially when capacity plateaus despite intensive training.

In children with CP, gait performance is mostly assessed by self- or proxy-report questionnaires which are inherently biased by subjectivity and misrepresentation (Capio et al., 2010). Since the advent of new assessment tools like accelerometer-based pedometers, actimeters or, more generally, inertial measurement units (IMU) based activity monitors, objective data can be collected from a patient’s daily activity, and direct comparisons with data measured in the laboratory can be performed.

Gait capacity and performance assessed by questionnaires are positively associated in children with CP (Van Eck et al., 2009), capacity exceeding performance (Young et al., 1996). However, this relationship is not constant in time and depends on the level of impairment [classified by the Gross Motor Function Classification System- GMFCS (Palisano et al., 1997; Ho et al., 2017]. Indeed, Van Gorp et al. (2018) have recently shown that performance keeps developing after the ceiling of capacity is reached. Studies using objective performance data have highlighted weak correlations of laboratory-based spatiotemporal and kinematic parameters with daily ambulatory activities of children with CP (Mitchell et al., 2015; Wilson et al., 2015; Guinet and Desailly, 2017; Nicholson et al., 2018; Wittry et al., 2018). Although the comparisons were based on dissimilar metrics (i.e., Gait Deviation Index (Schwartz and Rozumalski, 2008) versus step count per day, for instance), all these findings seem to indicate that gait performance cannot be predicted directly from gait capacity (Guinet and Desailly, 2017). On the other hand, we have recently demonstrated that gait in the laboratory was highly correlated with gait in daily life while comparing several identical metrics, including rhythm, pace, amplitude, stability, coordination, smoothness, variability and asymmetry metrics in children with CP (Carcreff et al., 2020). This study focused only on walking bouts in the real-world environment that approached those in the laboratory by their length and number of steps, but did not include the wide variety of bouts that take place during a usual day.

Walking speed is referred to as the sixth vital sign (Fritz and Lusardi, 2009) since it is a powerful indicator of mobility efficiency (Bjornson and Lennon, 2017; Van Ancum et al., 2019). This gait parameter constitutes the most reported outcome measure of interventions whose aim is to improve gait function (Bjornson and Lennon, 2017). It is an easy-to-administer objective and valid measure of walking activity that has been linked to functional ability and quality of life in children with CP (Moreau et al., 2016). It can reliably be estimated using IMUs, by direct integration, biomechanical modeling or machine learning methods (Yang and Li, 2012). We have recently demonstrated the satisfactory accuracy and reliability of speed estimation in children with CP with low to moderate levels of motor disability (GMFCS levels I to III), using a configuration of sensors on the shanks and thighs (Carcreff et al., 2018; Gerber et al., 2019). Recent studies have found that clinic-based speed corresponded to the highest speeds measured in daily conditions in community-dwelling older adults (Takayanagi et al., 2019; Van Ancum et al., 2019), in patients with Parkinson’s disease (Toosizadeh et al., 2015; Del Din et al., 2016a) and patients with multiple sclerosis (Supratak et al., 2018). This has not been explored in CP.

The aim of this pilot study was to compare the walking speed of youngsters with CP in the laboratory and in daily life, by employing our previously validated sensor configuration (Carcreff et al., 2018; Gerber et al., 2019) and maximizing the number of walking bouts from real life included into our analyses to reflect daily performance.

Analyses were also performed in healthy controls with typical development (TD) since daily-life assessments of gait function are novel in this population with a need for reference data in order to draw potential conclusions (Del Din et al., 2016b). Analyses were performed at the group level, and at the individual level, to account for the heterogeneity of the CP population.

Three specific research questions were formulated to allow this comparison: (1) Is spontaneous walking speed in the laboratory different from daily life?; (2) Is there an association between speed in the laboratory and in daily life?; and (3) How much does walking speed in the laboratory represent speed in daily life? Our hypotheses, based on the literature, were that: (1) walking speed is different in daily life and in clinical settings, (2) there is a moderate correlation between speeds estimated in these two contexts [as previously demonstrated (Mitchell et al., 2015; Wilson et al., 2015; Guinet and Desailly, 2017; Nicholson et al., 2018; Wittry et al., 2018)] and, (3) walking speed in the laboratory represents the highest speed in daily life for children with CP (as demonstrated in other populations).

## Materials and Methods

### Recruitment

Children, adolescents and young adults with CP (often referred to ‘children’ in the rest of the manuscript) were recruited for this pilot study from the pediatric orthopedic unit of Geneva University Hospitals using the following inclusion criteria: aged between 8 and 20 years, diagnosis of CP, ability to walk in the community with or without mechanical walking aids (e.g., crutches, tripods or walker), i.e., with a GMFCS level (Palisano et al., 1997) between I and III. A group of children, adolescents and young adults with TD, homogeneous in age and sex with the CP group, were recruited among collaborators’ or patients’ acquaintances. Individuals of both groups were excluded if they had additional impairments that limited their participation in the measurements (mental age <8 years, severe visual impairment, attention deficit and/or other significant behavioral issues). Written informed consent was obtained from participants ≥14 years old or their legal guardian if younger, and the protocol was approved by and carried out in accordance with the hospital’s institutional ethical committee (Cantonal Commission for Research Ethics of Geneva – CCER-15-176).

### Protocol

A trained investigator measured anthropometric data (shank and thigh lengths) and lower limb muscle strength. The GMFM (66-item version) (Alotaibi et al., 2014) was assessed for the children with CP by a trained evaluator to score their functional capacity (from 0 to 100, 100 being the highest capacity).

Children were equipped with wearable inertial sensors and reflective markers (optoelectronic system). They were asked to walk barefoot at a natural self-selected pace (with the instruction to walk “as usual, as you walk in the street”), back and forth on a 10-meter walkway within the gait laboratory, following a standard protocol of clinical gait analysis (Baker, 2013). A total of 4 to 10 trials were recorded for each participant, depending on their capacity and fatigue.

Next, the participants were asked to wear the sensors in their daily life, for a minimum of 10 waking hours per day, during 3 consecutive days including 2 week-days (school days) and 1 weekend day. The sensors were placed at the beginning and recharged at the end of each day of measurement by a parent, a caregiver or the participant him/herself. Previously, they received a practical training by the investigator on how to handle the sensors, i.e., turning on, fixing on body segments, turning off, charging, etc. A guide was also given to support them at home. The participants were asked to complete a diary reporting their physical activities and the eventual issues encountered with the sensors.

### Wearable Sensors

**FIGURE 1:**
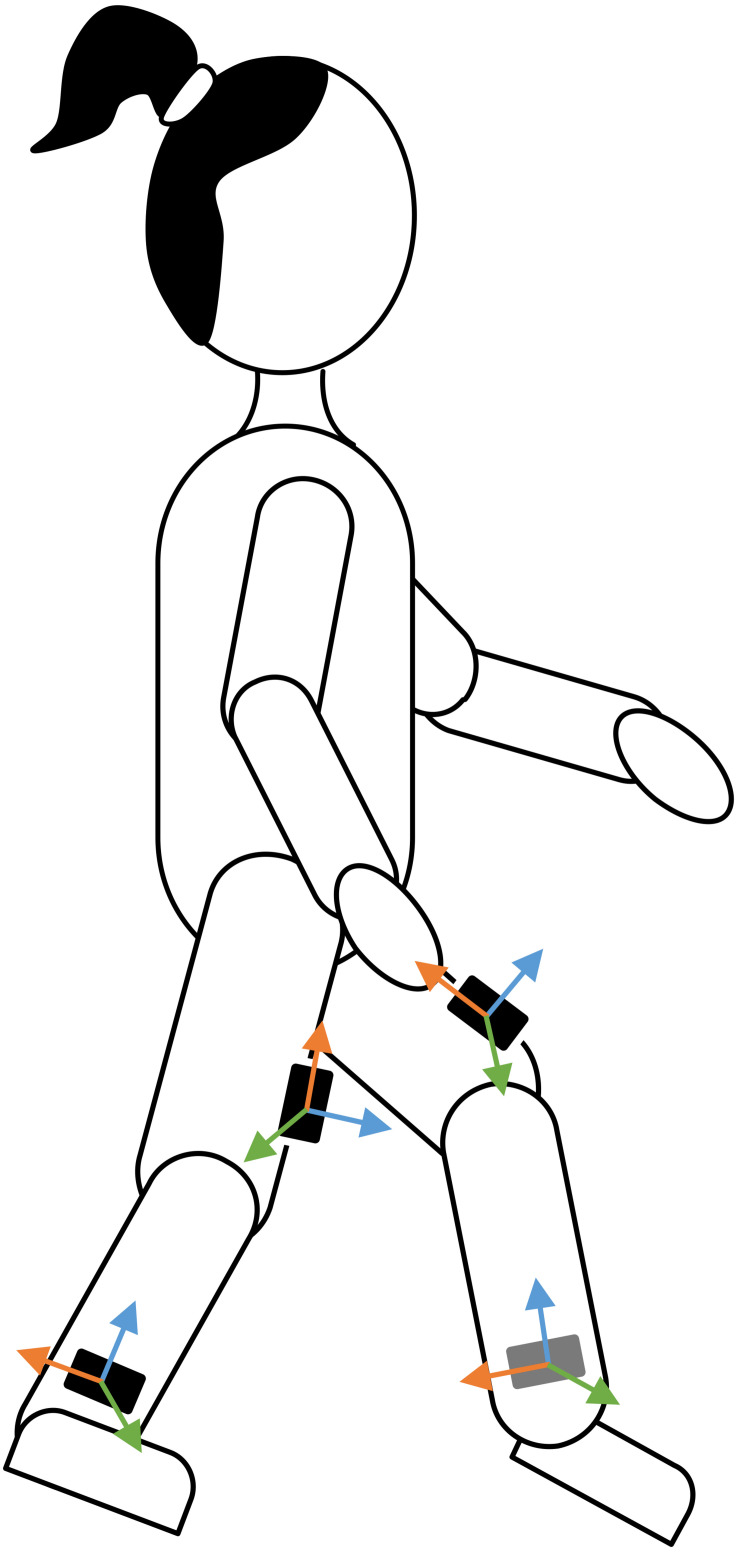
Sensor placement. Four sensors were placed on the participant’s lower limbs. The orientation of the sensors’ axes (colored arrows) was not imposed since the sensor-to-segment alignment was performed in post-processing using the PCA approach described in the section “Data Analysis.”

Four synchronized IMU-based devices (Physilog4^®^, Gait Up, Switzerland) were used, one on each shank and thigh (Figure 1), since this configuration was demonstrated to be the most adequate lower-limb configuration to assess the walking speed in youths with CP throughout GMFCS level I to III (Carcreff et al., 2018). Each device comprised a triaxial accelerometer (range ± 16 g) and gyroscope (range ± 1000°/s). The sampling frequency was set at 100 Hz. During the measures in the laboratory, the devices were safely fixed with a hypoallergenic adhesive film (Opsite Flexigrid, Smith & Nephew Medical, United Kingdom). During the daily life measures, the devices were fixed with a hypoallergenic double-sided hydrogel sticky (PAL stickies, PAL Technologies Ltd., United Kingdom) and protected from falling with a handmade Elastane sleeve, or under tight pants and socks.

### Optoelectronic System

The IMUs were synchronized with a twelve-camera optoelectronic system (Oqus7+, Qualisys Göteborg, Sweden) according to the protocol described in a previous paper whose goal was to compare gait parameter outputs from the clinical silver standard and the wearable system (Carcreff et al., 2018). In the present study, the optoelectronic system was only used to automatically crop continuous IMUs’ data into several straight gait trials (to discard turns and standing periods).

### Data Processing

**FIGURE 2:**
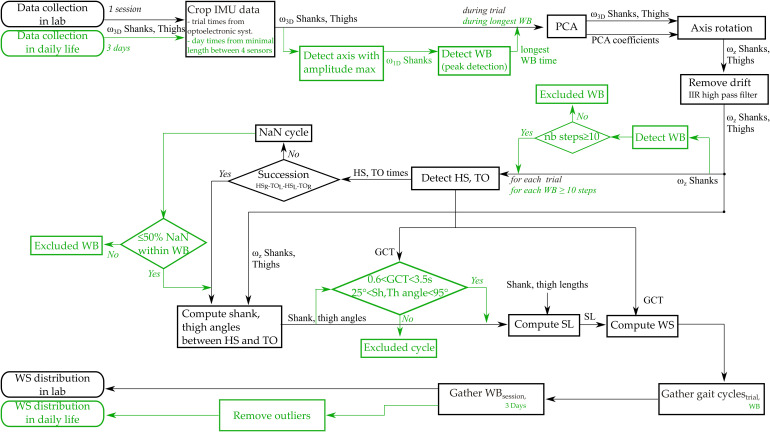
Flow chart of data processing steps. In green are represented the steps specific to daily life measures. Abbreviations: ω_1D_: 1-axis angular velocity; ω_3D_: 3-axis angular velocity; ω_z_: pitch angular velocity; PCA: Principal Component Analysis; WB: Walking bout; IIR: infinite impulse response; nb steps: number of steps; HS: Heel strike; TO: Toe off; HS_R_: Heel strike right; HS_L_: Heel strike left; TO_R_: Toe off right; TO_L_: Toe off left; NaN: Not A Number; GCT: Gait Cycle Time; Sh,Th: Shank and thigh; SL: Stride length; WS: Walking speed.

Figure 2 details the data processing steps.

#### In-Laboratory Measures

The 3D continuous angular velocity data was automatically cropped into several straight walking episodes (corresponding to each back and forth trial on the walkway in the laboratory, excluding the turns) by using the optoelectronic system’s start and stop of each trial. The pitch angular velocity (around the medio-lateral axis) was extracted using a principal component analysis (PCA), as the principal axis of movement during gait was assumed to correspond to the movement around the medio-lateral axis (Mcgrath et al., 2018). Gait events [heel strikes (HS), toe offs (TO)] were identified on the pitch angular velocity of the shanks as described by Aminian et al. (2002); Salarian et al. (2004). Succession of right and left steps were checked based on the times of HS and TO of each side. Shanks and thighs angles for each stride were computed by trapezoidal integration between HS and TO, and between TO and HS for each walking trial with good succession of right and left steps. Walking speed was computed (cf. “Walking speed computation” section) for each gait cycle and for each trial.

#### Daily Life Measures

Continuous recordings were cropped so that all files of the same day had the same time of recording (i.e., the minimal time shared between the 4 sensors). Walking episodes were detected using the axis of angular velocity with the highest amplitude of both shanks based on the peak detection method described by Salarian et al. (2004) personalized at the individuals level as described in a previous paper (Carcreff et al., 2019b). The 3D signal of the longest detected walking bout was used to determine the PCA coefficients to ensure selecting a steady-state walking bout. Then, the axis alignment was performed on the entire signal to extract the pitch angular velocity. Drift and noise on the signal were removed using an Infinite Impulse Response (IIR) high pass filter (Salarian et al., 2004). Only the episodes with a minimum of 10 steps were selected for further analysis. This threshold was chosen based on inputs from clinicians and was used in previous studies (Prajapati et al., 2011; Brodie et al., 2016). A minimum of 10 steps also approximately corresponded to the number of steps per 10 m trials in the laboratory while it would assure to have a continuous walking bout without break, less affected by the context such as short walking in a room, fidgeting, or obstacle avoidance. HS and TO were identified, the succession of right and left steps were checked, and the shank and thigh angles were computed for each stride as described in the “In-laboratory measures” section. Atypical values of gait cycle potentially related to environmental contexts (e.g., turning, obstacle avoidance) were excluded. Criteria for exclusion were defined by taking the minimum and maximum values of each parameter from previous in-laboratory assessments at various speeds, in both the CP and the TD groups, i.e., a gait cycle time less than 0.6 s and more than 3.5 s, or a shank sagittal ranges of motion less than 25° and more than 95° (Carcreff et al., 2019b, 2020). This procedure was necessary to exclude the false positive detected cycles. Stride length and walking speed were estimated for each remaining gait cycles of the included walking episode.

#### Walking Speed Computation

**FIGURE 3:**
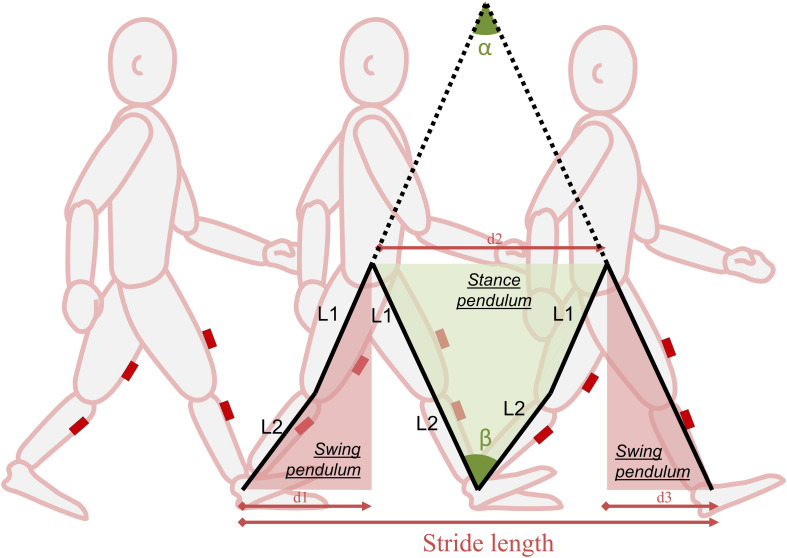
The double pendulum model. The double pendulum model estimates the stride length from the distances d1, d2, d3. d1 and d3 are estimated from α, which is obtained from the angular velocity signal of the thigh (during the swing pendulum) and the segments length (L1, L2); d2 is estimated from β, which is obtained from the angular velocity signal of the shank (during the stance pendulum) and the segments length (L1, L2). For more information, refer to Aminian et al. (2002).

For both sources of measurements (in-laboratory and daily-life), walking speed was computed from the ratio between the stride length and the stride time. The stride time is the time difference between two successive foot-strikes. The stride length is computed from the pitch angular velocity of the shanks and thighs, based on the double pendulum model (Aminian et al., 2002; Salarian et al., 2004). This model uses the thigh and shank lengths and their rotation in the sagittal plane (computed by numerical integration of pitch angular velocities) between foot-strike and foot-off, to compute the stride length, as illustrated on Figure 3. The accuracy of the system for walking speed estimation is 0.07 m/s, in children and adolescents with and without CP regardless of the level of impairment (Carcreff et al., 2018). Walking speed was thus reported by stride (including right and left steps).

### Data Analysis

GMFM scores, muscle strength information and use of walking aids were used as descriptive clinical features.

#### Description of the Ambulatory Contexts

The ambulatory activities (number of steps in-laboratory, total number of steps per day in daily life, median and maximal number of consecutive steps) were described for each group and each day type (week-day or weekend day) in order to set the context of the measurements. Cumulative Distribution Function (CDF) plots were used to visualize the speed distribution in-laboratory (CDF_Lab_) and during each day of daily life (CDF_DL_) on the same figure, in order to make direct visual comparisons between the distributions. CDF plots were preferred to histograms for better readability in the superposition of the distributions.

#### Comparisons Between Laboratory and Daily Life Speeds

The results obtained from the 3 days recorded data were gathered in order to provide a more accurate representation of gait throughout the week. A single daily life distribution representing both week-days and weekend days was thus obtained. Outlier values (∈[Q1-1.5^∗^IQR:Q3+1.5^∗^IQR] with Q1:1st quartile; Q3:3rd quartile and IQR: interquartile range) were excluded from this single distribution. The level of significance for the following statistical tests was set at *p* < 0.05.

#### Difference Between Speed in Laboratory and Speed in Daily Life

Median of speed distributions in laboratory and in daily life were compared for each group (group level) and subgroups of GMFCS using the nonparametric paired Wilcoxon tests (indicated for small sample size). The speed distribution (all gait cycles) in the laboratory was also compared to the daily life distribution for each participant (individual level) using unpaired Wilcoxon tests. Effect size was computed by dividing the Wilcoxon test statistic by the square root of the number of observations, as suggested by Pallant (2013). Results were described in absolute speed to guarantee better readability (by keeping meaningful units, i.e., m/s). Results regarding normalized speed [/√(g^∗^leg length) (Hof, 1996)] were also computed to ensure consistency of the conclusions.

#### Association Between Speed in Laboratory and Speed in Daily Life

The correlation between median speed in the laboratory and median speed in daily life was assessed by using Spearman’s rank correlation coefficient (rho). Altman’s guidelines were used to interpret the correlation as: poor if rho < 0.2; fair if 0.21 < rho < 0.40; moderate if 0.41 < rho < 0.60; good if 0.61 < rho < 0.80; and very good if rho > 0.81 (Altman and Altman, 1999).

#### Speed in the Laboratory Representative of Speed in Daily Life

The percentile from the daily life distribution that corresponded to the median speed in laboratory was calculated, according to the method of Van Ancum et al. (2019). As an example, if the median speed in daily life equals the median speed in laboratory, the corresponding percentile would be 50.

## Results

### Description of the Study Population and the Ambulatory Contexts

Fifteen youths with CP and 14 with TD were included in this study. The details about the CP population are provided in Table 1 and the characteristics of each group are presented in Table 2.

**TABLE 1:** Description of the CP population.

**Age**	**Sex**	**GMFCS**	**GMFM**	**Topography**	**More affected side**^1^	**Walking aid in laboratory**	**Walking aid in DL**
13.7	F	I	86.5	Uni	L		
12.3	F	I	89.7	Uni	R		
15.6	M	I	88.0	Uni	R		
20.1	M	I	88.0	Bi	R		
13.2	M	I	100.0	Bi	L		
12.3	F	I	100.0	Bi	R		
12.8	M	II	78.3	Bi	L		
10.3	F	II	65.6	Bi	R		wheelchair for long distances
9.3	F	II	67.4	Bi	L		
14	F	III	68.5	Bi	L		walker
15.8	F	III	54.1	Bi	R	walker	walker or wheelchair
8.3	M	III	57.9	Bi	R	walker	walker
11.3	M	III	54.9	Bi	L	walker	walker
11.6	F	III	63.6	Bi	L	crutches	crutches or wheelchair
17.5	F	III	37.4	Bi	R	walker	walker

*^1^: based on lower limb muscle strength testing; Uni: Unilaterally affected; Bi: Bilaterally affected; R: Right; L: Left; DL: Daily life, GMFCS: Gross Motor Function Classification System; GMFM: Gross motor function measure.*

**TABLE 2:** Groups’ characteristics and detected ambulatory activity.

	**CP**	**TD**
**Group characteristics**		
Sample size	15	14
Sex (*n* and % of girls)	9 (60%)	8 (57%)
Age (year)	12.8 [11.4:14.8]	12.2 [11.5:14.5]
Body mass (kg)	45 [36:53.5]	45.8 [37.8:57.0]
Body height (m)	1.53 [1.40:1.60]	1.57 [1.47:1.67]
**Laboratory**		
Number of steps	38 [28:47]	42.5 [31:46]
**Daily life**		
Number of steps per day^1^		
Week-days	5471 [4665:6930]	7343 [6364:9266]
Weekend days	4059 [3581:5248]	5583 [5086:6394]
Number of consecutive steps per day^1^		
Week-days	287 [136:499]	725 [532:987]
Weekend days	23 [22:25]	22 [21:26]
Maximal number of consecutive steps^1^		
Week-days	377 [154:612]	930 [744:1146]
Weekend days	143 [107:275]	376 [222:553]
Time detected walking per day (min)^1^		
Week-days	54 [24:62]	69 [58:86]
Weekend days	28 [23:32]	45 [38:58]

*^1^: Considering walking bouts with a minimum of 10 steps; value are medians [IQR] per group. CP: Cerebral palsy; TD: Typical development.*

For participants with CP, the dominant clinical pattern was spastic diplegia (*n* = 12). Therefore, the majority was affected on both sides, and half of the patients needed a walking aid for outdoor walking. The GMFM scores ranged between 37.4 and 100. One child with CP – GMFCS III did not walk enough during 2 days since she largely used her wheelchair to move, so no walking episode with more than 10 steps was detected, hence only 1 day (week-day) was considered for this participant. One adolescent with TD forgot to wear the sensors during the third day (weekend day). Two children with CP (GMFCS I and III) did not follow the instruction to wear the sensors on a weekend day, thus 3 week-days were assessed for them. Table 2 gives an overview of the detected ambulatory activities in each group in laboratory and daily life. In general, children with CP took fewer steps than children with TD. The number of steps taken, as well as the time spent walking during weekend days was lower compared with the week-days for both groups. Especially, the median number of consecutive steps during weekend days represented only 8 and 3% of the median number of consecutive steps during week-days in children with CP and TD, respectively. One child with TD took more than 10’000 steps and this during a week-day.

**FIGURE 4:**
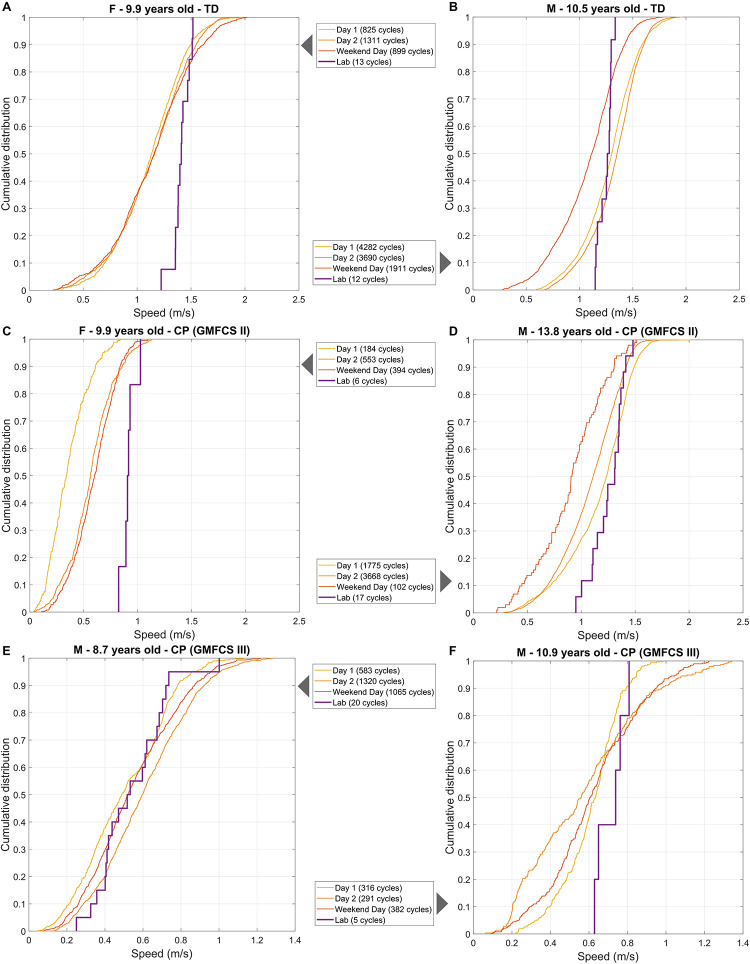
Six examples **(A–F)** of speed distributions (as Cumulative Distribution Function plots) in laboratory (‘Lab’) and 3 days of daily life: 2 week-days (“Day 1,” “Day 2”), and 1 weekend day. Abbreviations: CP: Cerebral palsy; TD: Typical development; F: Female; M: Male; GMFCS: Gross Motor Function Classification System.

The distributions of in-laboratory and daily-life speeds were visualized as superimposed CDF plots, and 6 examples are reported in Figure 4. We observed heterogeneous behaviors regarding the difference between in-laboratory and daily-life distributions. The right shift of the CDF_Lab_ with respect to the CDF_DL_ indicates that some children walked mainly at lower speeds in daily life than in laboratory (A, C and F cases). Others (like B, D and E cases) walked at similar speeds in both environments (CDFs are aligned or centered). Besides, the difference between the 3 days of daily life was not homogeneous in all participants. For instance, the child A had a similar speed distribution between the 3 days, whereas child B walked slower during the weekend.

### Comparisons Between Laboratory and Daily Life Speeds

Only the results regarding absolute speed were reported since normalized speed provided the same results.

#### Difference Between Walking Speed in Laboratory and Speed in Daily Life

**FIGURE 5:**
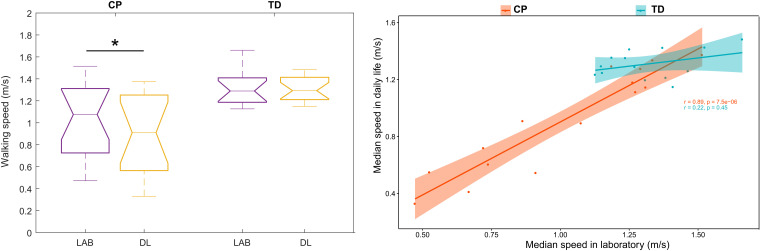
Comparison (left) and association (right) between median speed in the laboratory and daily life at the group level. ^∗^ stands for significant difference (*p* < 0.05). Abbreviations: LAB: Laboratory; DL: Daily life; CP: Cerebral palsy; TD: Typical development.

**FIGURE 6:**
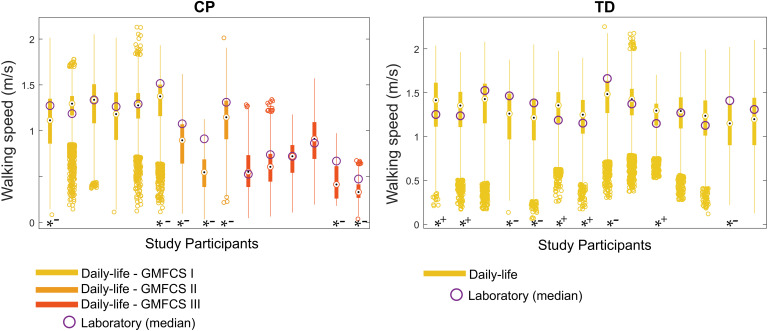
Intra-subject comparisons between laboratory and daily life. Daily life speed distribution is represented by the box plots. Stars at the bottom of the box plots stand for significant difference between distributions, tested by unpaired Wilcoxon tests (^*–^: Lab > Daily life; ^*+^: Lab < Daily life) for each study participant. Abbreviations: CP: Cerebral palsy; TD: Typical development; GMFCS: Gross Motor Function Classification System.

In the CP group, the median speed in daily life (3 days assembled) was significantly slower than the median speed in laboratory [Table 3 and Figure 5 (left)]. This was not the case for the TD group, for which the median speeds were similar. Furthermore, no significant difference was found when the comparison was performed for each subgroup of GMFCS (Table 3). Figure 6 illustrates the comparison between the median speed in laboratory and the distribution of daily life speeds for each participant (individual level). Proportions of individuals are summarized in Table 4. The main observations were that none of the children with CP or TD showed a median in-laboratory speed lower than the first quartile of daily life speed. Nine out of 14 (64.3%) participants with TD presented a significantly different [lower (*n* = 4) or higher (*n* = 5)] speed in daily life as compared to the laboratory. In the CP group, the participants who showed significantly lower speeds in daily life (*n* = 7, 46.7%) were equally distributed among the GMFCS levels: 2 with GMFCS I, 3 with GMFCS II and 2 with GMFCS III.

**TABLE 3:** Median speed (m/s) in the CP and TD groups in the two different measurement settings.

	**Laboratory**	**Daily life**	**Comparisons^*w*^**			**Correlations^*s*^**		**Corresponding percentile**
	**Median [IQR]**	**Median [IQR]**	***p*-Value**	**ES**	**95%CI**	***p*-Value**	**rho**	**Median [IQR]**
CP (*n* = 15)	1.07 [0.73–1.28]	0.91 [0.58–1.23]	**0.015**	0.543	[0.02–0.16]	**<0.001**	0.89	60 [50:75]
GMFCS I (*n* = 6)	1.28 [1.26–1.32]	1.29 [1.21–1.33]	0.313	0.141	[−0.11–0.16]	0.242	0.60	57.5 [51.3–63.8]
GMFCS II (*n* = 3)	1.07 [0.99–1.19]	0.89 [0.72–1.02]	0.250	0.275	[0.16–0.36]	0.333	1.00	75 [75–85]
GMFCS III (*n* = 6)	0.69 [0.56–0.73]	0.58 [0.45–0.69]	0.313	0.141	[−0.05–0.26]	**0.033**	0.89	62.5 [46.3–82.5]
TD (*n* = 14)	1.29 [1.20–1.40]	1.29 [1.24–1.40]	0.715	0.082	[−0.10–0.10]	0.454	0.22	42.5 [35:68.75]

*CP: Cerebral palsy; TD: Typical development; GMFCS: Gross Motor Function Classification System; IQR: Interquartile range; ES: Effect size; 95% CI: Confidence Interval; rho: Spearman’s correlation coefficient. ^*w*^: Paired Wilcoxon test; ^*s*^: Spearman’s rank correlation; Values in bold: significance (*p* < 0.05).*

**TABLE 4:** Proportion of individuals in the categories comparing speed in laboratory and speed in daily life.

	**In-lab median speed < [DL speed range]**	**In-lab median speed ∈ [DL speed range]**	**In-lab median speed > [DL speed range]**	In-lab speed significantly different from DL speed (*)
	
	**Proportion of individuals in the group (%)**			
CP (*n* = 15)	0.0	66.7	33.3	46.7
TD (*n* = 14)	0.0	85.7	14.3	64.3

*DL: Daily life; In-lab: in-laboratory; CP: Cerebral palsy; TD: Typical development.*

#### Association Between Speed in Laboratory and Speed in Daily Life

A significant very good correlation was found between the median speed in laboratory and in daily life for the CP group (rho = 0.89, *p* < 0.001) [Table 3 and Figure 5 (right)]. No correlation was found for the TD group (rho = 0.22, *p* < 0.454). A very good correlation was found for the GMFCS III-subgroup only (rho = 0.89, *p* = 0.033).

#### Speed in Laboratory Representative of the Speed in Daily Life

For the CP group, the speed in the laboratory represented the 60th percentile of the speed distribution in daily life (Table 3). Thus, the majority of children with CP walked at a lower speed during unsupervised walking. For the TD group, the corresponding percentile was 42.5, meaning that the speed in laboratory was between the 40th and the 45th percentiles of the daily life distribution of speed. Children with TD walked mostly at a higher speed during unsupervised walking. The median corresponding percentile for the GMFCS-level subgroups were 57.5, 75, and 62.5 for GMFCS I, II, and III, respectively.

## Discussion

The aim of this study was to evaluate the feasibility to compare in-laboratory and daily-life walking speeds in children with CP using wearable sensors, in order to gain new insights regarding the difference between gait capacity and performance in this population. Walking speed was considered to be a good indicator of the overall gait function (Middleton et al., 2015) and knowing its distribution over 3 real-life days revealed feasible and relevant to highlight the difference between the global level of performance and capacity. At the group level, children with CP showed a lower walking speed in daily life as compared to the laboratory. This was not the case for children with TD. Nevertheless, at the individual level, children with CP and with TD showed highly heterogeneous behaviors, and also among the GMFCS-level subgroups. Furthermore, in contrast to controls, a high correlation was found between median speeds in laboratory and daily life for children with CP (for the whole CP group and the GMFCS III subgroup). The speed adopted by children with CP during supervised walking corresponded mostly to their higher speeds in daily life: 60% of their daily walking activity was at a lower speed than in the laboratory. On the contrary, the speed adopted by the group of children with TD during supervised walking corresponded to the lower speeds in daily life: 60% of their daily walking activity was at a higher speed than in the laboratory.

### Considerations at the Group Level

The results found for children with CP, in line with those of our previous study assessing multiple gait characteristics (Carcreff et al., 2020) are not in total accordance with previous studies that used dissimilar metrics to compare gait performance with gait capacity. The significant slower walking speed in daily life as compared to the clinical environment was expected. It can be the result of several, and most probably, combined reasons. Firstly, the so called ‘Hawthorne effect’ (doing better when observed) (Berthelot et al., 2011) may have been verified here: children with CP walked mostly faster during the clinic-based assessments. Secondly, the external factors present in the real-life environment may have played an important role in decreasing gait velocity during daily life measurements. Indeed, slowing down could reflect the difficulty when dealing with uneven surfaces and obstacles (Toosizadeh et al., 2015), decreased concentration (Prajapati et al., 2011; Brodie et al., 2016), and also fatigue and cognitive-motor interferences which are two major difficulties associated with CP (Brunton and Rice, 2012; Katz-Leurer et al., 2014; Carcreff et al., 2019a). However, with our study participants, we could not draw any conclusion regarding the effect of fatigue on the walking speed, as illustrated in Supplementary Figure 1, especially given the few data for later hours. The high correlation between in-laboratory and daily life gait speeds was unexpected though. We would have expected that the wider variety of walking bouts included in the present analysis would provide different results as compared to our previous study (Carcreff et al., 2020), hence would better fit the low to moderate correlations found in the literature (Guinet and Desailly, 2017; Gosselin et al., 2018; Wittry et al., 2018). Nonetheless, our results suggested that, for children with CP, capacity is associated to and exceeds performance (Young et al., 1996). Considering the results found in each GMFCS level, this might be especially true for the more affected children (GMFCS III) even though the small sample size in each subgroup made difficult to draw definitive conclusion. This is in contrast with the behavior of healthy controls, and probably higher functioning children with CP, for whom no correlation and no significant differences were found. This can be explained by the difference in speed ranges for children with CP and TD. Indeed, higher range of speeds in CP favored better correlation. This also meant that the capacity-performance relation can be in both directions for TD: underperforming or outperforming their capacity in daily life. This can be attributed to their ability to vary their pace depending on the context, i.e., to ‘respond to unpredictability’, as described by Gosselin et al. (2018). Children with CP have less motor and attentional reserve for adaptability than their TD peers (Houwink et al., 2011; Gosselin et al., 2018), which may lead to quasi-systematic lower performances.

Recent studies which addressed this ‘lab. versus free-living’ clinical issue in adult and elderly populations agreed that clinic-based gait parameters were higher, and actually corresponded to the highest level of natural walking, i.e., to the ‘best performance’ (Brodie et al., 2016, 2017; Van Ancum et al., 2019). Even for high functioning people, the time spent walking at the level (speed or cadence) of in-laboratory walking is rare (Tudor-Locke et al., 2013). Our findings were not fully aligned with these statements.

### Considerations at the Individual Level

The results described at the group level need to be interpreted with caution since heterogeneous behaviors were found for individuals with CP, as well as those with TD, nuancing the previously mentioned group results. We found that only 46.7% of children with CP had a significant difference between supervised and unsupervised walking speed, while at the group level, the median speeds were highly significantly different. This is likely due to the small sample size and the high inter-subject variability. Indeed, the corresponding percentile ranged between 25 and 95, picturing completely different inter-subject behaviors. For children with TD, 64.3% did not follow the group results and the corresponding percentile ranged between 25 and 80. These intra-group heterogeneities are most likely due to dissimilar day-to-day lifestyles and dissimilar demonstrations of the spontaneous in-laboratory gait. Indeed, children’s daily activities are very variable. Depending on the school program, physical activities highly differ between days. Also, external factors, such as the weather, were inherently not controlled during the unsupervised assessments. The protocol should have included more days of measurement, as suggested by Ishikawa et al. (2013) (a minimum of 6 days for the higher functioning children, and 4 days for the most affected children), to obtain more stable measures of habitual ambulatory activity. A compromise had to be found though, to decrease the risk of patients’ refusing to wear the sensors in their daily environment, as encountered for three children of our cohort. Moreover, family situations, geographic location, and physical activity habits vary significantly across participants. All these factors could have contributed to the heterogeneity of the results. They could be further integrated into a multivariate analysis. In addition, the behaviors in laboratory may differ among children. First, although we used modified verbal commands (Nascimento et al., 2012) to instruct for ‘spontaneous’ speed (“walk as usual, as you were in the street”), the understanding of the instruction may have differed. A demonstration or a systematic training phase to let the participant find his spontaneous speed could have been considered (Nascimento et al., 2012). Second, the Hawthorne effect may have resulted in two different outcomes: walking faster or improving the gait pattern quality to the detriment of walking speed.

It should be noted that the heterogeneity within the CP group was not the result of the heterogeneous levels of impairments, since different results were found for the same gross motor function (GMFCS and GMFM) levels (Figure 6). This was in line with previous questionnaire-based results showing that for the same levels of capacity, different performances were observed (Holsbeeke et al., 2009). Accounting for the described individual heterogeneity, this study brought new evidence, based on objective assessments, to the relevance of assessing both capacity and performance for children and youths with CP. Even if the performance was highly associated with laboratory-based walking, the exact level of an individual’s performance cannot be predicted from the laboratory.

### Description of the Ambulatory Contexts

The description of the participant’s ambulatory activities showed big differences between the groups. The number of steps taken by children with CP and TD was about 5’000 and 7’000, respectively, during week-days, and 4’000 and 6’000 during weekend days. This was in agreement with the results of Bjornson et al. which showed that children with TD take more steps than children with CP on a daily basis (Bjornson et al., 2007). Furthermore, this indicates that our study participants did not reach the recommendations of 10’000 to 15’000 steps per day for children and adolescents (Tudor-Locke et al., 2011), with the exception of one child with TD. Several studies have pointed out this issue, which can be explained by the current tendency of children and adolescents to be more sedentary because of screen-based activities (Wu et al., 2017). However, since the focus of the current study was not to quantify physical activity but rather to qualify walking performances within meaningful walking bouts, many short walking bouts (<10 steps) were excluded from the analysis. In natural walking, most walking bouts are short (Orendurff et al., 2008) and we most likely overestimated sedentary behaviors by excluding short bouts.

### Clinical Implications

Nowadays almost all lab-based performance measures such as standardized tests, including 3D gait analysis, serve clinical-decision making, when real-life outcomes are actually the most important for the children and their families. The extent to which gait characteristics measured during 3D gait analysis correlates with unconstrained daily-life walking is still unknown (Supratak et al., 2018). It is fundamental to bridge this gap in order to appreciate the meaningfulness of clinic-based measures for the patient (Supratak et al., 2018). This study has the merit to address this issue thanks to walking speed, a clinically meaningful parameter considered as the sixth vital sign (Fritz and Lusardi, 2009), in opposition to arbitrarily defined levels of physical activity through ‘activity counts’.

This study emphasized that ecological assessments of gait should be considered in clinical routine, as a complement to in-laboratory 3D gait analysis, to obtain realistic information about motor performance, i.e., to capture an additional construct of the gait function (Van Ancum et al., 2019). This information about gait performance is highly valuable for clinicians when devising a treatment plan that can integrate widely varying elements from an intensive therapy program (Halma et al., 2020), through orthotics (Lintanf et al., 2018) to multilevel lower limb surgery (Amirmudin et al., 2019). Thanks to these assessments, clinicians will be able to verify that the effect of a treatment generalizes into the child’s daily life. Alternatives to the direct assessment of gait performance in daily life conditions, such as dual-task walking (Katz-Leurer et al., 2014; Carcreff et al., 2019a), or walking in semi-standardized or virtual reality environments (van der Krogt et al., 2014) can also be considered. Ultimately, the regular integration of objective day-to-day performance measurements as outcomes in the long term monitoring of treatments in children with CP, may allow better adjustment to individual needs and increase the personalization of motor management. As an example, with the here presented IMU measurement, the impact of adaptations of the patient’s environment on his performance could eventually be monitored and, based on the results, be adapted.

### Feedbacks on the Feasibility of a 3-Days Real-Life Gait Recordings

Although good acceptability of the measures was reported by participants and their families overall, daily-life assessments entail potential pitfalls. Indeed, a number of issues related to long-term measurements were reported in the diary or identified after data collection. The participants did not always follow the instructions to wear an Elastane sleeve or tight pants or socks to cover the sensors. The sensor fixation (PAL stickies, PAL Technologies Ltd., United Kingdom) alone was not sufficient. This problem was reported in 27% of the cases and was generally fixed by the participants with additional medical tape provided by the investigator. This should have been recommended from the beginning, as suggested previously (Del Din et al., 2016c). In the cases where the sensor fell and was re-placed in a different orientation (i.e., visible by a change in the signs of the signals), the PCA calibration was repeated for the corresponding part of the data. Surprisingly, no issue was reported by the parents or the caregivers who were in charge of handling the sensors every evening and morning. Errors were found a posteriori, such as interruption of the measurement before reaching 10 h of recording (7h50 in the worst case) most probably due to the child’s timetable (13.1% of the recordings), and bad switch-off at the end of the day (3.6% of the recordings). Finally, 9.5% of the measurements were interrupted because of battery loss of at least one sensor (after 6h30 of recording in the worst case). In any case, data was cut at the minimal time shared between the 4 sensors, resulting in an average of 11 ± 2 h of analyzed recordings per day. Globally, the feasibility of such assessments has been confirmed but several improvements need to be carried out, such as the sensor fixation, to enhance the usability of IMUs.

### Study Limitations

Additional study limitations need to be acknowledged. First, the sample size was low, only 29 participants, which is why this pilot study aimed at giving a first methodological framework to assess daily life performance rather than drawing definitive clinical conclusions for the CP population. However, the effect found in the CP group for the comparison between in-laboratory and daily life median speeds showed a medium substantial difference (effect size = 0.543) (Sullivan and Feinn, 2012) which is satisfactory for confidence in these preliminary results. Besides, limits related to the use of inertial sensors should be mentioned. Firstly, the calibration method based on PCA is not the most accurate approach from a biomechanical point of view. This method was adopted as an optimal solution since an approach based on functional calibration (Favre et al., 2009) using a pre-defined set of movements was difficult to be envisaged for children with functional disabilities, especially in the home environment without the supervision of the investigator. The PCA method is based on the assumption that the pitch angular velocity is maximal in the sagittal plane during forward walking. This assumption may have induced potential errors, with an impact on the computation of shank and thigh angles, hence on the walking speed estimation (Aminian et al., 2002), especially for the children with a high level of impairment, with higher frontal and transverse components at shank and thigh levels during walking. Further analyses should be undertaken to find a method for sensor-to-segment alignment that is accurate and feasible in this challenging population and at home. This would enable the computation of lower limbs kinematics, which are highly relevant gait features. Second, precautionary measures were applied to avoid the inclusion of non-walking activity into the analysis. However, we cannot exclude erroneous inclusion of false positives, which can be responsible for the outliers in the speed distribution (Figure 6). Furthermore, the double pendulum model proposed by Aminian et al. (2002) relies on precise leg dimension (thigh and shank segment lengths) measurements that can be challenging with patients with bone deformities and joint contractures (Sabharwal and Kumar, 2008). This was also a potential source of errors.

Last but not least, while walking speed in laboratory is estimated under same controlled conditions, walking speed in real-life condition is affected by the context changing the locomotion, e.g., due to crowd, weather, or path properties (Wang and Adamczyk, 2019). Information about this context of walking in daily life was not available as this is difficult to obtain in real-world condition unless the use of an additional system such as GPS (for location, indoor, outdoor) (Wang and Adamczyk, 2019) or an embedded camera (Hickey et al., 2017). However, the use of such additional devices is problematic in our population due to their age and privacy issue. As an attempt to limit the effect of the context, only walking bouts longer than 10 steps were included. However, the power law distribution of walking bouts (Orendurff et al., 2008), involves much more short walking bouts in daily life corresponding mostly to indoor walking or walking in a room, and 10 steps may represent more than the maximum number of steps taken in a row in the laboratory. Some solutions could be to include only frequently repeated walking bouts to eliminate unique behaviors or events from the analysis (Wang and Adamczyk, 2019); to use technological developments such as multimodal sensing (e.g., GPS, barometric pressure, microphone, weather records, etc.) to be more precise regarding the contexts, e.g., discriminate between indoor and outdoor, even and irregular surface, or straight and curved path, detect load carriage, a surrounding crowd or weather conditions (Wang and Adamczyk, 2019).

## Conclusion

This pilot study revealed that the assessment of walking speed in real-life conditions through IMU-based wearable sensors worn on the shanks and thighs was feasible and relevant to highlight the differences between a young individual’s performance and his capacity. Furthermore, results showed that walking speed was slower during natural walking as compared to laboratory-based walking in the group of children with CP. Speeds were also highly correlated which means that these children tended to under-use their gait capacity during daily life walking. In contrast, no difference was found between supervised and unsupervised walking in controls. Nevertheless, highly heterogeneous behaviors were observed at individual levels in both groups, and within GMFCS level sub-groups, which indicated that gait performance cannot be directly estimated from gait capacity. Overall, this study emphasizes the relevance of assessing natural walking as a complement to current capacity evaluations, and gives some clues about how to practically do it. Both assessments bring different and complementary information, which are valuable for clinicians, in the process of treatment planning and follow-up care.

## Data Availability Statement

The raw data supporting the conclusions of this article will be made available by the authors, without undue reservation.

## Ethics Statement

The studies involving human participants were reviewed and approved by the Cantonal Commission for Research Ethics of Geneva – CCER-15-176. Written informed consent to participate in this study was provided by the participants or their legal guardian.

## Author Contributions

CN, SA, GD, and AP-I contributed to the funding acquisition and conceptualization. AP-I, LC, CG, SA, and CN performed the methodology. LC, GD, CG, and SA contributed to the recruitment. LC and CG investigated the study. KA and AP-I performed the resources. LC and AP-I analyzed the software. LC contributed to the data curation, visualization, formal analysis, and writing-original draft. SA and KA supervised the study. SA, CN, and KA contributed to the project administration. All authors wrote, reviewed, and edited the manuscript.

## Conflict of Interest

CN and KA are advisory board members of Gait Up SA. The remaining authors declare that the research was conducted in the absence of any commercial or financial relationships that could be construed as a potential conflict of interest.

## Abbreviations

CP: cerebral palsy
F: female
GMFCS: Gross Motor Function Classification System
M: male
TD: typical development.
