# Supplementary material for: Walking Speed of Children and Adolescents With Cerebral Palsy: Laboratory Versus Daily Life
Source: Front Bioeng Biotechnol. 2020 Jul 14;8:812. doi: 10.3389/fbioe.2020.00812 (PMC7381141; doi:10.3389/fbioe.2020.00812)
Supplement: Supplementary file 1 [file Data_Sheet_1.PDF]

Supplementary Material

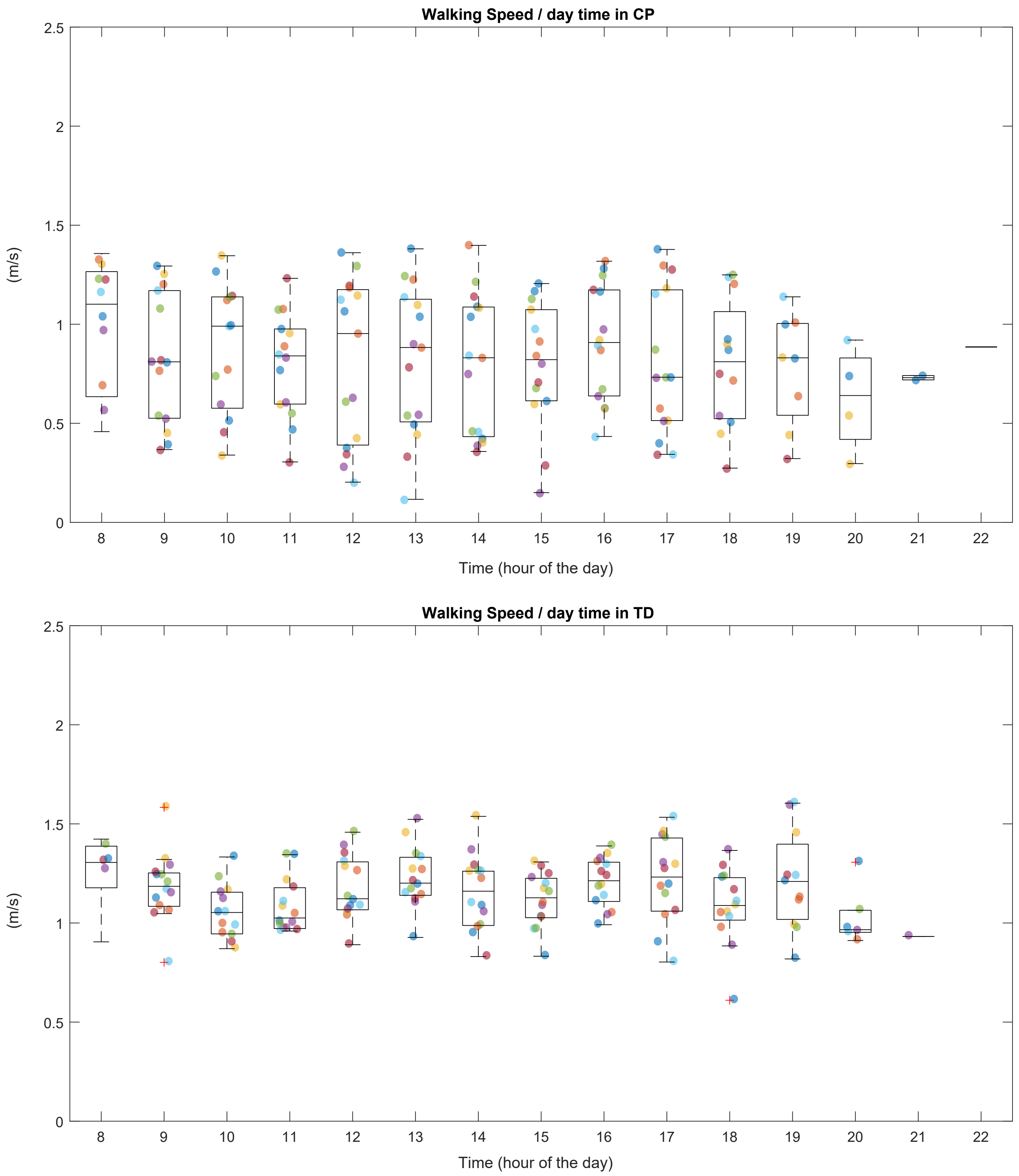

Supplementary Figure 1. Boxplots representing the mean walking speed per hour of the day for the groups of young individuals with cerebral palsy (CP) and typical development (TD). Each coloured dot represents a participant.
